# X-ray irradiated cultures of mouse cortical neural stem/progenitor cells recover cell viability and proliferation with dose-dependent kinetics

**DOI:** 10.1038/s41598-020-63348-2

**Published:** 2020-04-16

**Authors:** Valerio Licursi, Silvia Anzellotti, Jessica Favaro, Serena Sineri, Nicoletta Carucci, Enrico Cundari, Mario Fiore, Giulia Guarguaglini, Simone Pippa, Paola S. Nisi, Fiammetta Vernì, Stefano Biagioni, Emanuele Cacci, Roberto Amendola, Giuseppe Lupo, Rodolfo Negri

**Affiliations:** 1 Department of Biology and Biotechnology “C. Darwin”, Sapienza University of Rome, Rome, Italy; 2 Institute of Molecular Biology and Pathology, National Research Council (CNR), Rome, Italy; 3 Technical Unit for Radiation Biology and Human Health UTBIORAD, ENEA, Rome, Italy

**Keywords:** Developmental neurogenesis, Developmental neurogenesis, Neural stem cells, Neural stem cells

## Abstract

Exposure of the developing or adult brain to ionizing radiation (IR) can cause cognitive impairment and/or brain cancer, by targeting neural stem/progenitor cells (NSPCs). IR effects on NSPCs include transient cell cycle arrest, permanent cell cycle exit/differentiation, or cell death, depending on the experimental conditions. *In vivo* studies suggest that brain age influences NSPC response to IR, but whether this is due to intrinsic NSPC changes or to niche environment modifications remains unclear. Here, we describe the dose-dependent, time-dependent effects of X-ray IR in NSPC cultures derived from the mouse foetal cerebral cortex. We show that, although cortical NSPCs are resistant to low/moderate IR doses, high level IR exposure causes cell death, accumulation of DNA double-strand breaks, activation of p53-related molecular pathways and cell cycle alterations. Irradiated NSPC cultures transiently upregulate differentiation markers, but recover control levels of proliferation, viability and gene expression in the second week post-irradiation. These results are consistent with previously described *in vivo* effects of IR in the developing mouse cortex, and distinct from those observed in adult NSPC niches or *in vitro* adult NSPC cultures, suggesting that intrinsic differences in NSPCs of different origins might determine, at least in part, their response to IR.

## Introduction

Eukaryotic cells respond to genotoxic damage by several regulatory mechanisms leading to cell cycle delay, to the activation of DNA repair systems, to a complex reprogramming of gene expression involving many different protection circuits and, in the case of irreparable damage, to the onset of cell senescence or apoptosis^1,2^. The relative intensity of these different effects depends on several variables: the cell type and its physiological state; the extent and quality of the genotoxic insult; the timing of the insult relative to the cell cycle dynamics. Ionizing radiation (IR) is one of the major sources of genotoxic stress for human cells, due to its diagnostic and therapeutic use and to involuntary exposure^3–5^. The response of mammalian cells to IR of different quality and dose has been deeply analyzed and showed to be highly heterogeneous in different cell types and tissues^6–8^. In particular, there is an increasing interest among the biomedical scientific community in the response of stem cells to IR. This is related to two relevant considerations: (i) stem cells niches are often present in normal tissues exposed to off-target radiation during radiotherapy protocols, and predicting the consequences of this irradiation is crucial to improve therapy design^9^; (ii) stem cells are the best model to understand the mechanisms underlying the delicate balance between radioresistance and radiosensitivity, with the former favouring accumulation of genetic instability in the surviving cells and the latter causing stem cell depletion along with the clearance of genotoxic damage.

Pluripotent stem cells, such as embryonic stem cells, although showing high capacity to prevent and repair genetic lesions^10^, are generally very sensitive to genotoxic damage in general^11–13^, and to ionizing radiation in particular^14^, exhibiting higher rates of apoptosis than those observed in differentiated cells^15^. In contrast, adult stem/progenitor cell populations, even though very heterogeneous in their response to genotoxic damage, appear generally more resistant than pluripotent stem cells, possibly due to a peculiar DNA damage response^16^, which is functional to their role in promoting the reconstitution of damaged tissues and organs^17–19^. Among the features associated with the differences in radiosensitivity of various stem/progenitor cell populations, cell cycle length has been positively correlated with radioresistance, while the duration of p53 response and the intensity of mitochondrial priming have been considered sensitizing factors due to their apoptosis promoting action^12^. An increased radioresistance, if not balanced by appropriate surveillance mechanisms, could lead in principle to increased genomic instability. Therefore, it is crucial to understand the mechanisms of radioresistance for those adult stem cell niches particularly exposed to off-target radiation during radiotherapy, such as those harbouring neural stem/progenitor cells (NSPCs).

Murine NSPC cultures derived from the adult brain have been shown to be particularly radioresistant, showing a low mortality, following X-ray irradiation^20,21^. To explain this remarkable radioresistance, these studies propose a model of radiation-induced differentiation/senescence, in which the genotoxic insult causes permanent cell cycle exit and terminal differentiation of irradiated NSPCs. This process would increase cellular resistance to genotoxic damage, while avoiding cell death and the transmission of genomic instability. Similar observations have been reported for NSPCs derived from *in vitro* conversion of pluripotent stem cells^20^. This model is partially consistent with the results of *in vivo* irradiation of the adult mouse brain, which causes both apoptosis and terminal differentiation of proliferating NSPCs^22^, but is less congruent with the effects of *in vivo* irradiation of the foetal and neonatal mouse brain, which leads to NSPC apoptosis, followed by the recovery of proliferation by the surviving NSPCs^22–25^. In this study, we have investigated the dose-dependent and time-dependent response of NSPC cultures derived from the mouse foetal cerebral cortex to X-ray irradiation. We show that, within hours of high dose irradiation, cortical NSPCs undergo DNA damage and upregulation of p53 pathway genes, leading to cell death, cell cycle alterations and a transient upregulation of differentiation markers in the first few days after irradiation. In the second week post-irradiation, however, NSPC cultures recover control levels of p53-related transcripts, viability and proliferation, in the absence of detectable differentiation. These observations are in line with the previously described *in vivo* effects of irradiation in the developing cerebral cortex and suggest that the response of NSPCs to IR might be intrinsically affected by their age and/or regional identity.

## Materials and Methods

### NSPC culture and irradiation

This work was carried out by *in vitro* culture of available liquid nitrogen stocks of mouse NSPCs that were previously derived from the cerebral cortex of embryonic day 13.5 (E13.5) embryos. The original derivation of mouse cortical NSPCs was performed in accordance with EU and Italian regulations and with ethical approval by the Ethical Commitee for Animal Research of the Italian Ministry of Health, as described^26,27^. No additional animals were employed for the experiments reported in the present study. NSPC culture in adherent proliferating conditions was performed according to published protocols^26,27^. For routine expansion, cells were seeded in T25 flasks (Corning) coated with 10 µg ml^−1^ poly-ornithine (Sigma-Aldrich) and 5 µg ml^−1^ laminin (Corning) at a density of 10000–20000 cells/cm^2^, using previously described chemically-defined media^26,27^ supplemented with 20 ng/ml human recombinant Epidermal Growth Factor (R&D systems), 10 ng/ml human recombinant Fibroblast Growth Factor-basic (Peprotech), 1/100 N-2 supplement (Invitrogen) and 1/100 ITS supplement (Invitrogen). NSPCs were passaged every 3 to 4 days using Accutase (Corning). NSPCs expanded for not more than 25 passages since their initial derivation were used for this work. For irradiation experiments, cells were seeded 2 days earlier and media were replaced 30 minutes before treatment. Cultures were irradiated with 0.2 Gy, 1 Gy and 10 Gy of X-rays using a MLG 300/6 Gilardoni device with a dose rate of approximately 0.7 Gy/minute. Sham treated cultures were kept near the Gilardoni device for the same amount of time without exposure to X-rays. For analyses at 4 hours (4 h), 8 h and 24 h post-irradiation, cultures were harvested at the desired time point without media replacement. For analyses at the 48 h time point, media were replaced at 24 h post-irradiation. For analyses at 8 days (8d) after irradiation, sham treated and 1 Gy irradiated cultures were passaged twice at 48 h and at 5d to 7d post-irradiation, those treated with 10 Gy IR were passaged once at 5d to 7d post-IR. For differentiation assays, sham treated and irradiated cultures were maintained as above. Following passaging at 7d post-irradiation, half of the cultures were switched to differentiation media on the next day, media were replaced 3 days later and cells were harvested for real-time RT-PCR analysis or fixed for immunocytochemistry after 5 days since the start of differentiation. The remaining half was maintained in proliferating conditions and harvested usually 3 days after seeding. Differentiation media had the same composition of proliferation-supporting media, except that N2 and ITS supplements were replaced with 1/50 B27 Plus supplement (Invitrogen) and EGF was replaced with 25 ng/ml human recombinant Brain Derived Neurotrophic Factor (Peprotech). Independent experimental replicates were performed using different batches of NSPC cultures seeded in different dates. In each experiment, sham and X-ray treated NSPCs were seeded in parallel from the same NSPC batch. The number of independent experiments for each assay is indicated in the figure legends.

### Cell viability and cell cycle assays

To assess cell viability, adherent NSPC cultures at the desired time point were detached with Accutase, and recovered in their previous culture medium to include non-attached cells in the analysis. A 90 µl aliquot of this cell suspension was mixed with 10 µl of 0.4% trypan blue solution (Invitrogen) and used to count the fraction of non-viable, trypan blue-positive cells using disposable Glasstic slides (Kova International), according to manufacturer instructions.

For cell cycle analyses, sham and X-ray treated NSPCs at the desired time point were recovered as described above and centrifuged to obtain a cell pellet, which was rinsed with PBS, followed by fixation in cold PBS-methanol 1:1 and storage in this solution at 4 °C for a few days to a few weeks. After centrifugation and a rinse with PBS, cells were resuspended in PBS containing 50 µg/ml propidium iodide, 100 µg/ml RNase A and 0.1% Triton X-100. The fraction of cells in G1, S and G2/M, based on propidium iodide labelling of DNA content, was determined by flow cytometry on an Epics XL (Beckman Coulter) apparatus equipped with a Laser at 488 nm wavelength excitation. For each sample, 10^4^ events were collected, followed by the analysis of monoparametric histograms of DNA content by means of WinMDI 2.9 software.

### Real-time RT-PCR

Total RNA was extracted from frozen cell pellets using the miRNeasy Mini Kit (Qiagen) and quantified with a NanoDrop 1000 spectrophotometer (Thermo Scientific). For each RNA sample, 1 µg of total RNA was reverse-transcribed using the SensiFast cDNA Synthesis Reverse Kit (Bioline) and amplified with a 7500 Fast Real-Time PCR System (Applied Biosystems), using the SensiFAST™ SYBR® LoROX Kit (Bioline) and MicroAmp® Optical 96-Well Reaction Plates (Applied Biosystems), according to manufacturer instructions. Relative quantification of transcript levels was carried out with the 2^(−ΔΔCt) method, using *Gapdh* as a reference gene for assays in proliferating conditions and the average Ct of *Gapdh*, *Rpl19* and *Rpl29* for differentiation assays. The sequences of the primers used for real-time RT-PCR assays are reported in Table S1 in the Supplementary Information.

### Immunocytochemistry

For immunofluorescence analysis of phosphorylated histone H2AX (γH2AX), NSPCs were plated on poly-ornithine/laminin coated glass coverslips in 24-well plates (Corning), then sham or X-ray treated as described above. At the desired time point, cells were fixed for 20 minutes with methanol-free formaldehyde (Pierce) diluted to 4% with PBS, then rinsed a few times with PBS and stored in PBS at 4 °C. For immunostaining, cells were treated with permeabilization solution (0.5% Triton X-100 in PBS) for 10 minutes, rinsed twice with PBS and incubated in blocking buffer (5% normal goat serum in PBS) for 1 h at room temperature, followed by overnight incubation at 4 °C with an anti-γH2AX (Ser139) (20E3) rabbit monoclonal antibody (Cell Signaling) diluted 1/200 in blocking solution. Cells were then rinsed twice with PBS and three times with 0.1% normal goat serum in PBS, followed by 90 minute incubation at room temperature with a goat anti-mouse IgG Cy3-conjugated antibody (Jackson Immunoresearch) diluted 1/200 in blocking solution. After rinsing with PBS, cells were incubated with Hoechst in PBS for 5 minutes at room temperature to label DNA, then rinsed again with PBS and mounted on glass slides with Dako fluorescence mounting medium. Samples were analysed using a Nikon Eclipse 90i microscope equipped with a 100x objective (oil immersion; N.A. 1.3) and a Qicam Fast 1394 CCD camera (QImaging). Images were acquired using Nis-Elements AR 3.2 (Nikon). Quantification of fluorescence signals was performed using the Nis-Elements HC 5.02 software (Nikon). Nuclei stained by Hoechst were identified with the Autodetect function. Automated measures were then performed within nuclei selections, to obtain the mean value of pixel intensity for the γH2AX signal for each object. Measured values were used for statistical analysis as described below.

For immunofluorescence analysis of NESTIN, GFAP and TUBB3, sham treated or irradiated NSPCs at 7d post-irradiation were plated on poly-ornithine/laminin coated glass coverslips in 24-well plates, cultured with proliferation-supporting or differentiation media, and fixed with 4% formaldehyde as described above. Immunostaining was then performed using mouse anti-NESTIN (Millipore, 1/200 dilution), rabbit anti-GFAP (Dako, 1/200 dilution) and mouse anti-TUBB3 (Promega, 1/400 dilution) primary antibodies, together with anti-mouse IgG Cy3-conjugated and anti-rabbit Alexa Fluor 488-conjugated secondary antibodies (both from Jackson immunoresearch, 1/300 dilution). Samples were analysed as above using a 40x objective.

### Statistical Analysis

Statistical analysis of experimental data was performed using Student’s t-test, or analysis of variance (ANOVA) followed by multiple comparison Tukey’s test, as indicated in the figure legends and in Table S2 in the Supplementary Information. Relevant p-values for the analysed experiments are reported in Table S2. All statistical analysis was carried out using native functions of R language version 3.5.3.

## Results

### Mouse cortex NSPC cultures survive moderate doses of X-rays and recover control viability levels in the second week after high dose irradiation

We previously described the derivation of NSPCs from the mouse cerebral cortex at E13.5, showing that, in agreement with other studies^28^, these cells can be efficiently expanded *in vitro* in adherent, chemically-defined culture conditions, retaining long-term ability for self-renewal and neuronal/glial differentiation, and a stable regional identity^26,27^. In the present work, we employed this experimental system to: (i) characterise the effects of different doses of X-ray IR on the viability, proliferation and DNA damage of NSPCs; (ii) investigate the transcriptional regulation of key molecular pathways associated with these effects; (iii) dissect the temporal kinetics of NSPC responses to IR.

**Figure 1:**
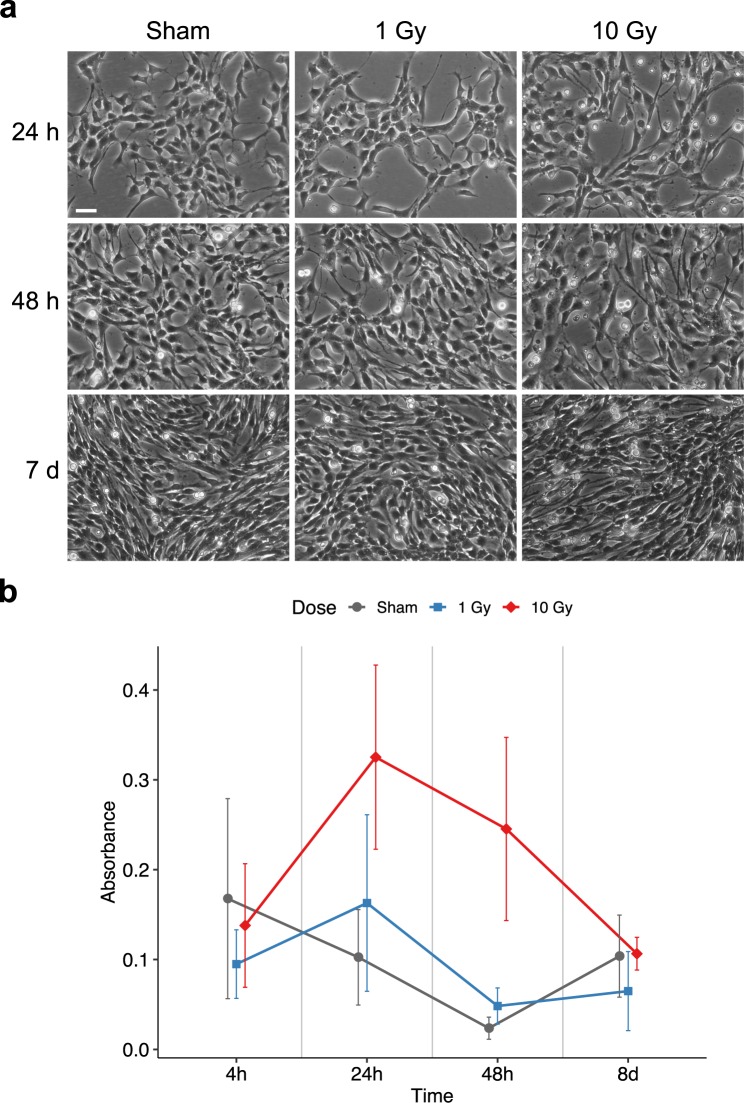
High doses of X-rays cause cell death of mouse cortex NSPCs, but the surviving NSPCs recover control viability levels in the second week post-irradiation. **(a)** Phase contrast images of sham treated (left), 1 Gy irradiated (middle) or 10 Gy irradiated (right) NSPC cultures at 24 h (top), 48 h (middle) or 7d (bottom) post-irradiation. In comparison with sham treated cultures, those irradiated with 10 Gy IR show reduced growth and increased debris due to cell death at the 24 h and 48 h time points, followed by a recovery at 7d. Scale bar, 40 μm. **(b)** Quantification of the fraction of trypan blue-positive cells at different time points (4 h, 24 h, 48 h, 8d) after sham treatment (grey line) or irradiation with 1 Gy (blue line) or 10 Gy (red line). No obvious differences are detectable between sham and 1 Gy samples. In comparison with these conditions, 10 Gy IR causes a marked increase of non-viable cells at 24 h and 48 h post-irradiation, which is recovered within a week. Dots in the chart represent the mean of 3 to 6 independent experiments. Error bars show the standard deviation. The statistical analysis of the data according to two-way ANOVA is reported in Table S2.

We first analysed the dose-dependent and time-dependent effects of X-ray IR on NSPC viability. Cortical NSPCs cultured in adherent conditions were irradiated 48 h after seeding with moderate (1 Gy) and high (10 Gy) doses of X-rays. Cell viability was quantified at different time points (4 h, 24 h, 48 h and 8d) post-irradiation by trypan blue exclusion staining, as described in the Methods section. At all these time points, the fraction of non-viable (trypan blue-positive) NSPCs in the irradiated cultures was compared with that found in sham treated cultures. As shown in Fig. 1a,b, sham treatments resulted in low levels of cell death throughout the time course (roughly 10% at 24 h and 8d). This is in line with the background cell death in NSPC *in vitro* cultures detected in other studies^29^, which is at least partially due to the enzymatic treatment needed to detach the cells before trypan blue analysis. The slightly higher fraction (roughly 15%) of trypan blue-positive cells observed in sham samples at 4 h post-irradiation may reflect a mild stress caused by keeping the cultures at room temperature and atmospheric CO_2_ during irradiation. No evident differences in cell viability were observed following 1 Gy irradiation when compared to sham treated cultures. In contrast, a 10 Gy dose of IR caused a marked increase in the fraction of non-viable cells both at 24 h and 48 h post-irradiation (Fig. 1b). Since the culture medium was changed at 24 h post-IR to get rid of dead cells, it is likely that a substantial amount of the trypan blue-positive cells detected at the 48 h time point were cells dying during the second day after irradiation. Furthermore, if the background levels of non-viable cells that are not due to IR (as detected in the sham samples) are subtracted from the levels observed in 10 Gy treated samples, a constant rate of IR-associated cell death (roughly 22% of the total cell count at each time point) appears to take place during the first and the second day following irradiation. Cell loss in the cultures treated with 10 Gy continued to be detectable during the first week post-irradiation (Fig. 1a and data not shown). Towards the end of the first week, however, irradiated NSPC cultures recovered and showed similar morphology, confluence and viability to sham treated cultures at 8d to 10d after irradiation (Fig. 1b; see below). These data suggest that moderate doses of X-rays (1 Gy) are sublethal for mouse cortex NSPCs, although we cannot rule out the possibility that a limited amount of cell death may detectable using more sensitive assays. High doses (10 Gy) cause partial cell death during the first week post-irradiation, which is recovered thereafter.

### X-ray irradiated mouse cortex NSPC cultures undergo dose-dependent cell cycle alterations, but recover control cycling profiles in the second week post-irradiation

**Figure 2:**
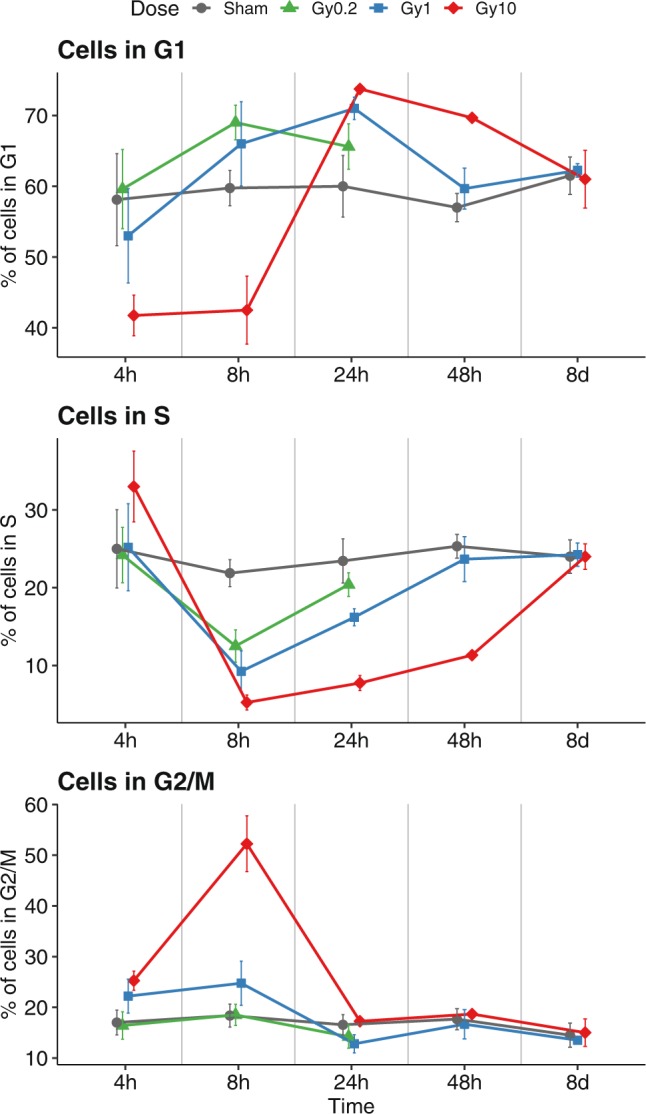
X-ray irradiation causes dose-dependent cell cycle alterations in mouse cortex NSPC cultures, which are recovered in the second week post-irradiation. Quantification of the fraction of cells in G1 (top), S (middle), G2/M (bottom) phases of the cell cycle at different time points (4 h, 8 h, 24 h, 48 h, 8d) after sham treatment (grey line) or irradiation with 0.2 Gy (green line), 1 Gy (blue line) or 10 Gy (red line). Irradiation causes dose-dependent effects, consisting in a decrease of the S phase fraction after 8 h to 48 h, an increase of the G2/M fraction after 8 h, and an increase of the G1 fraction after 24 h to 48 h. Sham-like profiles are recovered after 8d. Dots in the charts represent the mean of 3 to 9 independent experiments. Error bars show the standard deviation. The statistical analysis of the data according to two-way ANOVA is reported in Table S2.

We next studied the dose-dependent and time-dependent effects of X-ray IR on NSPC cell cycle. Adherent cortex NSPC cultures were irradiated 48 h after seeding with low (0.2 Gy), moderate (1 Gy) and high (10 Gy) doses of X-rays. Cell cycle analysis was performed at different time points (4 h, 8 h, 24 h, 48 h and 8d) post-irradiation by flow cytometry quantification of DNA content in cells stained with propidium iodide, as described in the Methods section, to estimate the fraction of cells in each phase of the cell cycle (G1, S, G2/M) in irradiated and sham treated cultures. As shown in Fig. 2, steady percentages of cells in G1 (roughly 60%), S (roughly 25%) and G2/M (roughly 15%) were detectable in sham samples throughout the time course (grey lines in Fig. 2). Low doses of X-rays (0.2 Gy, green lines in Fig. 2) caused a transient accumulation of cells in G1 at the expense of the S phase, which was observed at the 8 h time point and largely recovered by 24 h after irradiation. Given this early recovery, 0.2 Gy treated samples were not analysed beyond 24 h post-IR. In cultures treated with moderate X-ray doses (1 Gy, blue lines in Fig. 2), the accumulation in G1 was preceded by a slight increase in the G2/M cell fraction at the 4 h to 8 h time points and lasted longer, with a recovery of sham-like G1 and S percentages seen by 48 h post-irradiation. These effects were clearly enhanced in cultures exposed to high doses of X-rays (10 Gy, red lines in Fig. 2). A large increase in the G2/M fraction was found at the 8 h time point, with a corresponding decrease in the proportion of cells in the G1 and S phases. Even after 10 Gy IR, the effects on G2/M were transient and recovered to sham-like levels by 24 h post-IR. Similar to what observed with 1 Gy IR, the early increase in G2/M levels was followed by accumulation in G1 at the expense of the S phase. Altered cell fractions in the G1 and S phases were still detectable at 48 h after 10 Gy irradiation, but a recovery eventually ensued also at these high doses of IR, since the cell cycle profiles of irradiated and sham treated cultures were similar at 8d post-irradiation. These data suggest that X-ray irradiation alters the cell cycle dynamics of mouse cortex NSPC cultures, causing an early accumulation in G2/M within a few hours post-IR, followed by a slower accumulation in G1 during the next few days. They also indicate that the effects on the cell cycle are dose-dependent, causing stronger and longer changes in cell cycle distribution with increasing doses of X-rays, but recoverable even at high doses, in line with the results of cell viability assays.

### Clearance of the γH2AX foci induced by high dose X-ray irradiation in mouse cortex NSPC cultures

**Figure 3:**
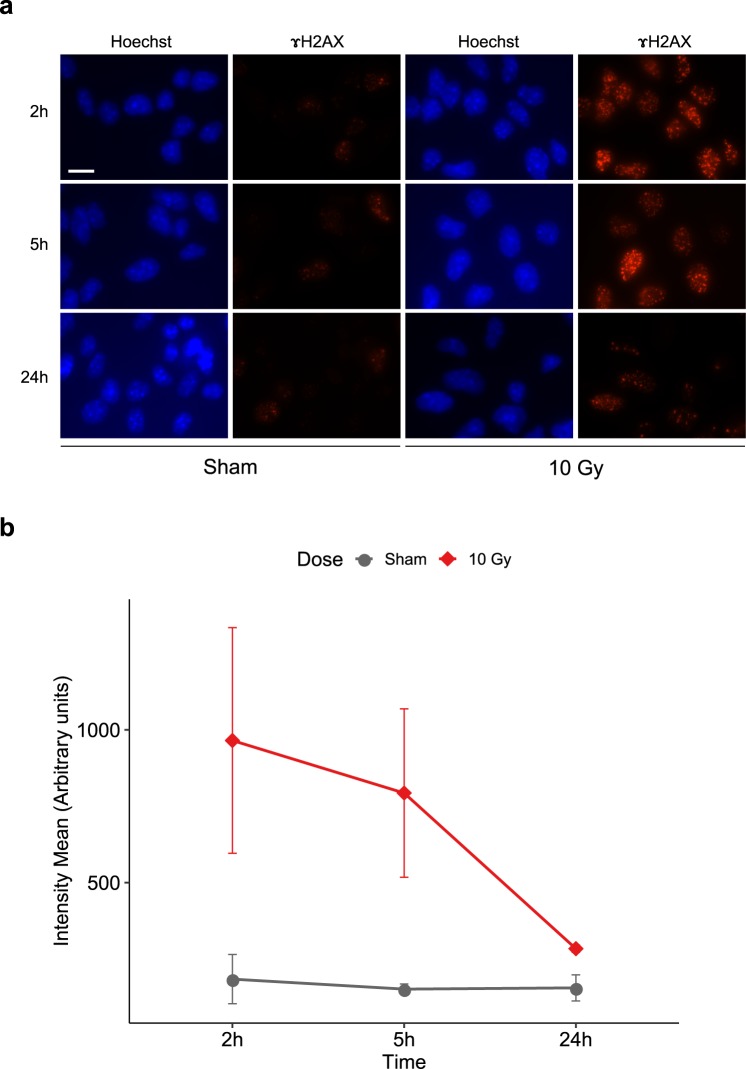
Accumulation of γH2AX foci in X-ray treated mouse cortex NSPC cultures is substantially cleared by 24 h post-irradiation. (**a**) Representative images of mouse cortex NSPCs that were sham treated or 10 Gy irradiated and immunostained with an anti-γH2AX antibody (red signal) at 2 h, 5 h or 24 h post-irradiation, as indicated. Nuclear Hoechst staining is shown in blue. Scale bar, 10 μm. **(b**) Quantification of the mean γH2AX fluorescent intensity per cell in each treatment condition and time point, performed as described in the Methods section. Dots in the charts represent the mean of 4 independent experiments. Approximately 50–100 cells per conditions were analysed in each experiment. Error bars show the standard deviation. The statistical analysis of the data according to two-way ANOVA is reported in Table S2.

The above results suggest that a proportion of irradiated cortical NSPCs alter their cell cycle in response to IR-dependent damage and undergo cell death if unable to repair the damage, or recover normal cell cycle dynamics in the case of a successful repair. This prompted us to examine the presence of DNA double-strand breaks, a typical consequence of IR, by means of immunofluorescence analysis with an antibody against γH2AX, a marker of the chromatin surrounding double-strand breaks. The average γH2AX nuclear signal in NSPCs was quantified at 2 h, 5 h and 24 h following 10 Gy irradiation or sham treatment, as described in the Methods section. Due to the fast kinetics of γH2AX foci accumulation and clearance in other types of NSPC cultures^20^, we limited this analysis to the first 24 h after irradiation and selected earlier time points (2 h and 5 h post-IR) than those used for cell viability and cell cycle assays (4 h and 8 h post-IR). Representative images of sham and irradiated cells at each time point are shown in Fig. 3a, and the quantitative analysis is reported in Fig. 3b. A clear increase in nuclear γH2AX levels, and in the number and intensity of γH2AX foci, was detectable at both the 2 h and the 5 h time points in irradiated cultures in comparison with sham treatments, followed by a marked recovery by 24 h post-IR. In agreement with previous *in vitro* studies on other NSPC types^20^, these results indicate that an extensive DNA double-strand break damage takes place in the majority of mouse cortex NSPCs exposed to a 10 Gy dose of X-rays. They also suggest that a proportion of cortical NSPCs can substantially repair this damage, and that the cells retaining elevated damage levels are eliminated through cell death.

### X-ray irradiation of mouse cortex NSPC cultures causes a transient, dose-dependent upregulation of p53 pathway genes and differentiation markers, which is recovered in the second week post-irradiation

The molecular networks regulated by the p53 tumour suppressor play crucial roles in the response of mammalian cells, including stem/progenitor cells, to DNA damage. Key elements of the p53-regulated, damage-associated, pathways include, for example, the cyclin-dependent kinase inhibitor CDKN1A (also known as p21), the pro-apoptotic mitochondrial protein BAX, and the stress-response protein GADD45, which is implicated in cell cycle arrest, DNA repair, and cell death. By upregulating the expression of the genes coding for CDKN1A, GADD45 and BAX, p53 promotes cell cycle arrest in response to DNA damage, followed by either a successful repair and cell cycle re-entry, or the activation of cell death pathways in the case of unsuccessful repair^30^.

**Figure 4:**
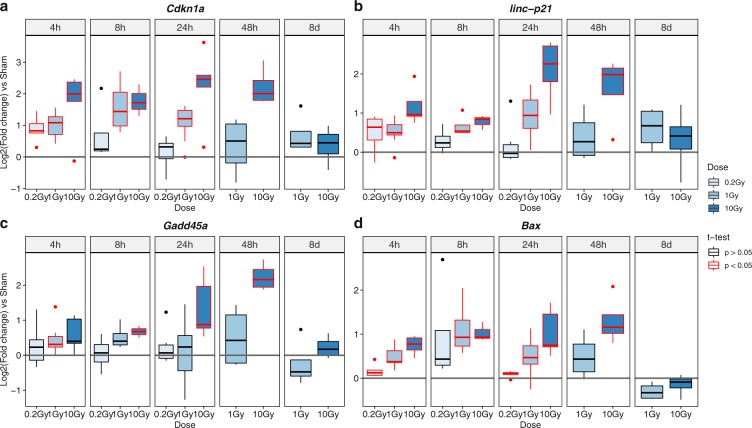
X-ray irradiation of mouse cortex NSPC cultures causes a transient, dose-dependent upregulation of p53 pathway genes. Box-and-whisker plots of the log_2_-transformed transcript levels of *Cdkn1a* **(a)**, *linc-p21* **(b)**, *Gadd45* **(c)** and *Bax* **(d)** in mouse cortex NSPC cultures irradiated with the indicated doses of X-rays, following real-time RT-PCR analysis and normalization to the sham treatment condition. Individual charts show the data collected at the indicated time points post-irradiation. Whiskers represent the distribution of the relative transcript levels for each gene at the indicated doses and time points. The lower and higher whiskers or dots indicate the minimum and maximum values, respectively. The bottom and top of the box represent the first and third quartiles, respectively, and the band inside the box indicates the second quartile (the median). Grey lines represent the relative expression levels in the sham condition. Plots show the results of 3 to 10 independent experiments. Plots highlighted in red indicate p < 0.05 for the comparison between irradiated and sham treated samples according to Student’s t-test.

Therefore, to confirm the recovery of irradiated cortical NSPC cultures at the molecular level, and to dissect in more detail the dose-dependent and time-dependent dynamics of the DNA damage response in these cultures, we employed real-time RT-PCR assays to compare the transcript levels of *Cdkn1a*, *Gadd45* and *Bax* in cultures exposed to 0.2 Gy, 1 Gy, 10 Gy IR or sham treatment. We also included the long non-coding RNA *linc-p21* among the analysed transcripts, since its expression levels are a sensitive readout of p53-dependent damage response pathways^31^. These assays were carried out at the same time points (4 h, 8 h, 24 h, 48 h and 8d post-irradiation) used for cell cycle assays, performing the analysis of 0.2 Gy treated samples until 24 h post-IR due to the recovery of sham-like gene expression profiles by this time point (see below). In comparison with sham samples, we observed dose-dependent transcriptional changes for *Cdkn1a* (Fig. 4a), *linc-p21* (Fig. 4b), *Gadd45* (Fig. 4c) and *Bax* (Fig. 4d) between 4 h and 48 h post-irradiation, with both the extent and the duration of the changes increasing in cultures exposed to higher levels of X-rays. For examples, for both *Cdkn1a* and *linc-p21*, low irradiation doses (0.2 Gy) caused a moderate upregulation at the 4 h time point, with a return to sham-like expression levels at 24 h post-irradiation. Moderate IR doses (1 Gy), led to a stronger transcriptional increase that persisted at the 24 h time point, but was recovered by 48 h after irradiation. Notably, high levels of X-rays (10 Gy) resulted in a coordinated upregulation of *Cdkn1a*, *linc-p21*, *Gadd45* and *Bax* that peaked at 24 h to 48 h post-irradiation, in agreement with the observed effects on cell viability and cell cycle profiles at these time points, but was recovered to sham expression levels by 8d after irradiation.

**Figure 5:**
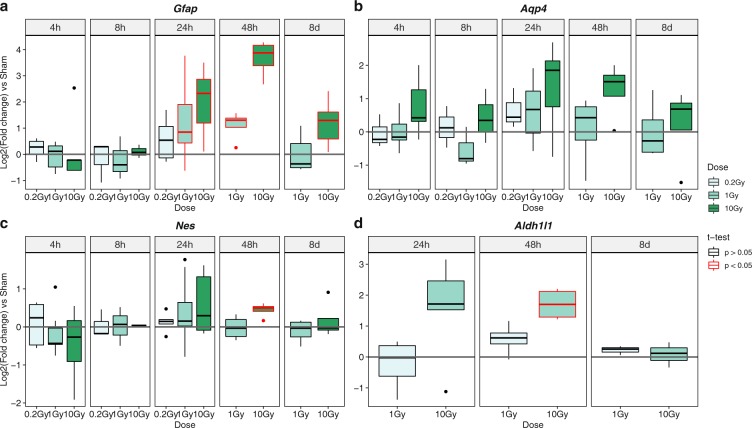
X-ray irradiation of mouse cortex NSPC cultures causes a transient, dose-dependent upregulation of astrocytic markers. Box-and-whisker plots of the log_2_-transformed transcript levels of *Gfap* **(a)**, *Aqp4* **(b)**, *Nes* **(c)** and *Aldh1l1* **(d)** in mouse cortex NSPC cultures irradiated with the indicated doses of X-rays, following real-time RT-PCR analysis and normalization to the sham treatment condition. Grey lines represent the relative expression levels in the sham condition. Plots show the results of 3 to 10 independent experiments. Plots highlighted in red indicate p < 0.05 for the comparison between irradiated and sham treated samples according to Student’s t-test.

Previous studies have suggested that IR can promote terminal differentiation of mouse NSPCs derived from pluripotent stem cells or from the adult brain, as a protective mechanism that prevents the proliferation of cells suffering DNA damage, at the same time avoiding extensive cell death^20,21^. This prompted us to monitor the transcript levels of three well established astrocytic markers, namely *Gfap*, *Aqp4* and *Aldh1l1*, in the same samples used for the expression analyses of p53 pathway genes. The results of these assays are shown in Fig. 5a (*Gfap*), Fig. 5b (*Aqp4*) and Fig. 5d (*Aldh1l1*). They revealed, at 24 h to 48 h after 10 Gy irradiation, an upregulation of all of the tested astrocytic genes in comparison with sham samples, with a stronger effect for *Gfap*. Milder changes were detectable following 1 Gy irradiation, whereas low doses of X-rays (0.2 Gy) caused no evident effects. Notably, the expression levels of *Gfap*, *Aqp4* and *Aldh1l1* declined beyond the 48 h time point, generally returning to sham-like levels at 8d post-irradiation. Although *Gfap* was still upregulated in 10 Gy irradiated samples compared to sham samples at the 8d time point, its transcript levels were reduced to roughly 25% of those detected at 48 h after irradiation, and they were similar to those of sham samples at later time points (see below). Similar results were obtained with the neuronal markers *Dcx* and *Tubb3* (also known as *βIII-Tubulin*) (Fig. S1; see below). These observations suggest that high doses of IR cause a transient upregulation of astrocytic and neuronal markers in cortical NSPC cultures, but do not lead, overall, to their terminal differentiation. This conclusion is supported by the stable, sham-like expression levels of the NSPC marker *Nestin* (*Nes*) in irradiated cultures throughout the analysed time course (Fig. 5c), and by the comparable morphology of sham treated and irradiated NSPCs in the first week after irradiation (Fig. 1a).

**Figure 6:**
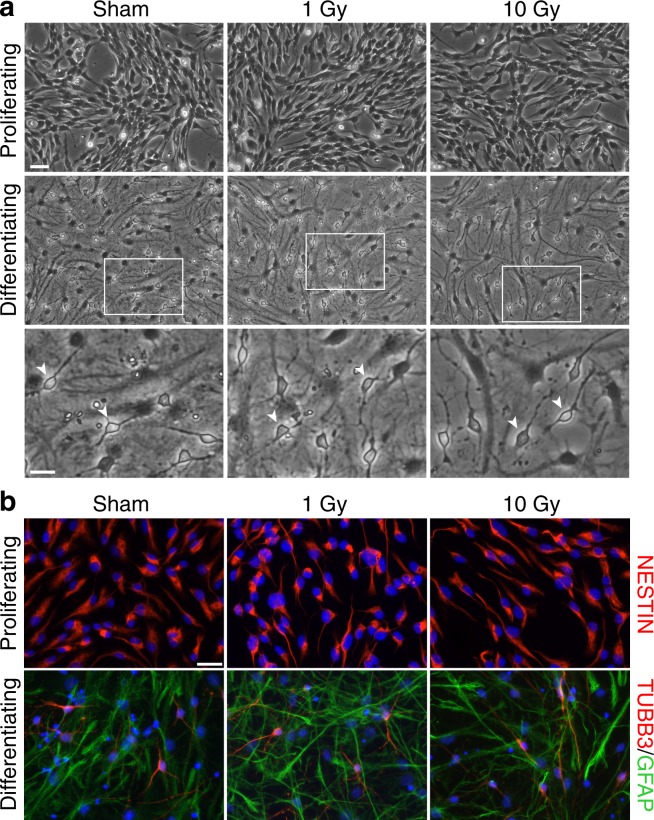
Mouse cortex NSPC cultures in the second week post-irradiation show a widespread differentiation in the absence, but not in the presence, of exogenous EGF. (**a**) Phase contrast images of sham treated (left), 1 Gy irradiated (middle) or 10 Gy irradiated (right) cortex NSPC cultures that were passaged at 7d post-irradiation and maintained in EGF-containing media for 3 additional days (top) or switched to differentiation media devoid of exogenous EGF for 5 days (middle and bottom). A widespread presence of differentiating neuronal and glial cells can be observed in the absence, but not in the presence, of EGF, both in sham treated and in irradiated cultures. White arrowheads point to some examples of differentiating neurons. The white boxes indicate the regions shown at higher magnification in the images below. Scale bars, 40 μm (top) and 20 μm (bottom). (**b**) Representative images of sham treated (left), 1 Gy irradiated (middle) or 10 Gy irradiated (right) cortex NSPC cultures that were cultured in the presence (top) or in the absence (bottom) of exogenous EGF and immunostained with an anti-NESTIN antibody (top, red signal) or a combination of anti-GFAP and anti-TUBB3 antibodies (bottom; red signal, TUBB3; green signal, GFAP). Nuclear Hoechst staining is shown in blue. Scale bar, 20 μm.

**Figure 7:**
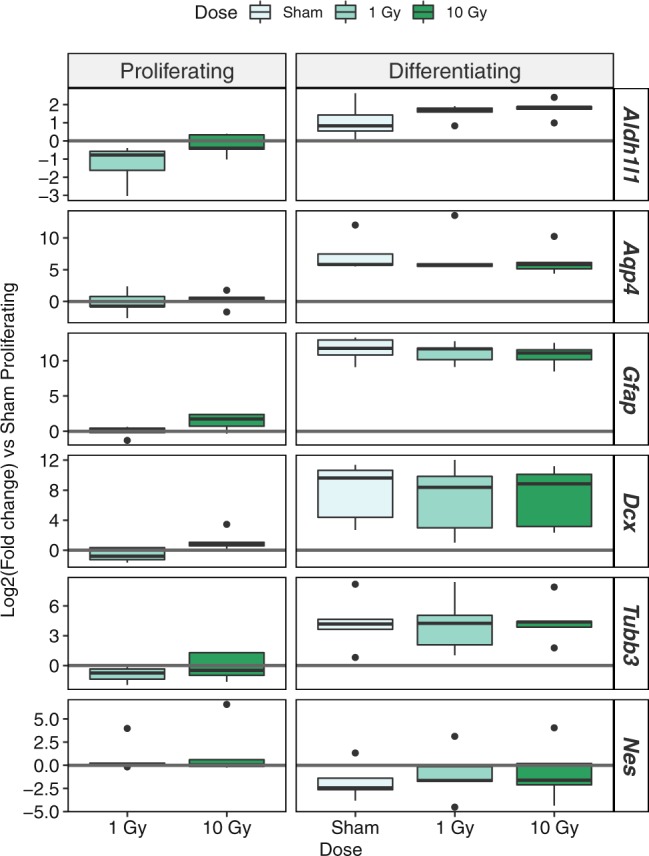
Sham treated and irradiated mouse cortical NSPC cultures in the second week post-irradiation show similar upregulation of astrocytic and neuronal markers in the absence of exogenous EGF. Box-and-whisker plots of the log_2_-transformed transcript levels of *Aldh1l1*, *Aqp4*, *Gfap*, *Dcx*, *Tubb3* and *Nes* in mouse cortex NSPC cultures irradiated with the indicated doses of X-rays and cultured in conditions supporting proliferation or differentiation, following real-time RT-PCR analysis and normalization to the levels in the sham proliferating condition (shown by the grey lines). Differentiating cultures show a clear upregulation of *Aldh1l1*, *Aqp4*, *Gfap*, *Dcx*, *Tubb3* and a limited downregulation of *Nes* with respect to proliferating cultures, with no major differences detectable between sham and X-ray treated samples. Plots show the results of 5 independent experiments.

To better address the effects of IR on NSPC differentiation, we extended these analyses to mouse cortex NSPCs that were induced to differentiate by culturing them in media devoid of exogenous EGF, as described in the Methods section. A week after 1 Gy or 10 Gy irradiation or sham treatment, NSPC cultures were passaged and either maintained in proliferation-supporting media containing exogenous EGF for 3 additional days, or switched to differentiation media for 5 days. As shown in Fig. 6a, cultures maintained in the presence of EGF nearly homogeneously retained an undifferentiated morphology regardless of the previous treatment condition, which was confirmed by immunostaining for the NSPC marker NESTIN (Fig. 6b). In contrast, both sham treated and irradiated NSPCs that were switched to media devoid of EGF acquired the typical morphology of differentiating neuronal and glial cells, which was confirmed by immunostaining for the neuronal marker TUBB3 and the astrocyte marker GFAP. Real-time RT-PCR assays showed a clear upregulation of astrocytic (*Aldh1l1*, *Aqp4*, *Gfap*) and neuronal (*Dcx*, *Tubb3*) genes in differentiating cultures in comparison with proliferating cultures, with no major differences between sham treated and irradiated samples (Fig. 7). Of note, the transient increase in the mRNA levels of these differentiation markers observed at 24 h to 48 h post-irradiation (Figs. 5, S1) was clearly lower than the increase promoted by differentiation media (Fig. 7). Altogether, these results indicate that cortical NSPC cultures in the second week of recovery from high dose irradiation largely maintain an undifferentiated phenotype in the presence of exogenous EGF, but are competent to differentiate to neurons and glia upon EGF withdrawal.

## Discussion

IR-induced DNA damage in stem/progenitor cells usually causes an early cell cycle arrest, but the subsequent events, including apoptosis, differentiation, senescence, and cell cycle re-entry, may be significantly different in distinct stem cell compartments^32^. In the case of NSPCs, several studies have investigated their response to IR both *in vivo* and *in vitro*, reporting heterogeneous and even contradictory findings. Notably, *in vivo* analyses in rodents have suggested that the specific response of NSPCs to IR-dependent DNA damage may depend, at least in part, on the age of the animal, possibly due to the known age-associated changes in the NSPC population occurring between foetal and adult stages^33,34^. In particular, NSPCs of the developing brain, such as those located in the ventricular and subventricular zones (VZ/SVZ) of the foetal cerebral cortex and in the VZ/SVZ lining the lateral ventricle in the neonatal brain, are generally highly proliferative and broadly sensitive to IR-induced apoptosis, but those NSPCs that survive irradiation resume proliferation and repopulate the NSPC niche^22–25^. In contrast, NSPCs of the adult VZ/SVZ niche can exist in an actively proliferating or in a quiescent status^34^. The former subpopulation is radiosensitive and responds to IR by undergoing apoptosis or terminal differentiation, in either case depleting the proliferating NSPC pool; the latter subpopulation is radioresistant and responds to IR by entering the cell cycle and replenishing the proliferating NSPC pool, although the response was shown to depend on the total dose and the dose fractionation scheme that were applied^22,35–39^. Irradiation of the NSPC niche *in vivo*, however, causes both cell-autonomous effects on NSPCs and non-cell-autonomous effects mediated by other, non-neurogenic, cell populations of the niche, and the direct and indirect influences of IR on NSPC fate are very difficult to distinguish. For example, it has been shown that IR can stimulate endothelial cells of the adult mouse VZ/SVZ to produce TGFβ1, which can cause cell cycle arrest and apoptosis of NSPCs. Importantly, these effects were observed when NSPCs from untreated mice were grafted in the VZ/SVZ of irradiated mice or co-cultured with irradiated endothelial cells, indicating that the irradiated niche non-cell-autonomously affects NSPC fate^39^. Therefore, it is important to complement *in vivo* analyses with *in vitro* studies on well defined NSPC cultures derived from specific brain regions and ages, in order to dissect the direct effects of IR on NSPCs, in the absence of indirect effects mediated by other cell populations.

**Figure 8:**
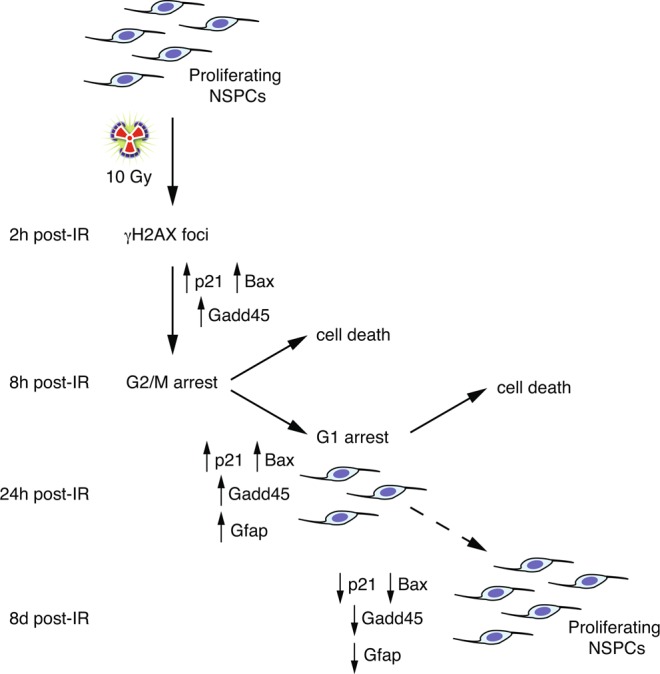
Proposed model of the time-dependent effects of 10 Gy irradiation in mouse cortex NSPC cultures. Exposure of mouse cortex NSPC cultures to high X-ray levels causes a rapid increase of γH2AX foci and p53 pathway gene expression levels within few hours, followed by an early cell cycle arrest in G2/M and a later arrest in G1. By 24 h post-irradiation, significant apoptosis and upregulation of differentiation markers (e.g. *Gfap*) ensues, but the surviving cells are able to mitigate DNA damage and the transcriptional changes in p53 pathway and differentiation genes, restoring normal viability and proliferation rates. The dashed arrow between 24 h and 8d indicate that a phenotypic recovery of irradiated NSPCs takes places between these time points, but the exact timing was not characterized. See text for further details.

In this work, we provide evidence that mouse foetal cortical NSPC cultures show several key responses similar to those observed in the context of *in vivo* studies. In proliferating NSPC cultures, IR causes an early accumulation in G2/M, followed by a later accumulation in G1 (Fig. 8), similar to what previously described *in vivo* for the irradiated mouse foetal cortex, although the timing of these events (measured as hours post-irradiation) is slower *in vitro* than *in vivo*^24,40^. The cell cycle alterations of irradiated cultures are accompanied by significant cell death, followed by the resumed proliferation of a proportion of the surviving cells and culture repopulation (Fig. 8). Although we detected a transient increase in the expression levels of differentiation markers at 24 to 48 h after irradiation, a morphological and molecular comparison of these cultures with those induced to differentiate by the withdrawal of exogenous EGF suggests that irradiation *per se* does not lead to a widespread differentiation of cortical NSPCs *in vitro*. Nonetheless, it is possible that, among the cells surviving irradiation, some exit the cell cycle and differentiate, but become diluted by proliferating cells and/or are eliminated through passaging. Further work will be needed to quantify the fraction of irradiated NSPCs that remain in the cell cycle and drive the post-irradiation recovery of the cultures.

The *in vitro* responses of cortical NSPC cultures are consistent with the effects observed following *in vivo* irradiation of the foetal mouse cortex and the neonatal VZ/SVZ^22–25,40^, but they are distinct from those described in the adult VZ/SVZ niche. In the latter case, the actively proliferating NSPC pool is wiped out due to the occurrence of both apoptosis and terminal differentiation, whereas the quiescent NSPC pool is insensitive to apoptosis and responds to IR by entering the cell cycle and repopulating the proliferating compartment^22,35–37^. Of note, *in vitro* NSPC cultures derived from the adult VZ/SVZ^20,21^, differently from foetal cortical NSPC cultures (this study), undergo wider IR-induced cell cycle exit and differentiation, suggesting that the distinct responses of different NSPC niches to IR may be partially recapitulated *in vitro*.

Although our *in vitro* results are generally consistent with *in vivo* data, they extend them in two ways. First, our assays include a higher dose (10 Gy) compared to what usually employed *in vivo* at foetal stages (2 Gy)^22,24,40^. Second, they specifically assess the intrinsic response of NSPCs to IR, without the extrinsic influences that are known to be mediated by the non-neurogenic cell types of the *in vivo* niche^39^. On the one hand, our data show that irradiated cortical NSPC cultures undergo apoptosis even when they are isolated from their *in vivo* niche. On the other hand, even after 10 Gy irradiation, a fraction of the irradiated NSPC population is able to repair the damage, avoiding cell death and differentiation, and reconstitute the proliferating NSPC pool. Our data also show that cortex NSPC cultures in the second week after irradiation remain competent to differentiate towards neuronal and glial fates under appropriate conditions, although more work will be needed to determine whether the efficiency of differentiation towards specific cell types and/or their functional maturation are affected by IR.

Taken together, the results of this study, along with previous reports^20–25,35–40^, suggest the following conclusions: i) apoptosis is an intrinsic response of cortical NSPCs to IR and is not solely due to alterations of the surrounding niche, although the latter can enhance IR-induced NSPC death; ii) the differential response of foetal and adult NSPCs to IR might be partially due to intrinsic differences in these NSPC populations and not solely to variations in the extrinsic environment of their niches, although the latter are also likely to play a role.

Building up on these conclusions, two closely related questions stand out for future investigations. First, it is unclear why foetal cortical NSPCs are not equally sensitive to IR and a fraction of them may avoid apoptosis and cell cycle exit. Second, the mechanisms underpinning the differential response of foetal vs adult NSPCs, and quiescent vs proliferating NSPCs, remain to be addressed. Although it has been suggested that the observed differences in NSPC response to IR are not due to their proliferative status *per se*^22^, we note that the amount of control NSPCs in S-G2/M closely matches the amount of non-viable NSPCs at 24 h post-irradiation. This suggests that NSPCs in the S-G2/M phase of the cell cycle may be more sensitive to IR, as previously suggested based on *in vivo* analyses^24,40^. Besides the proliferative status, other factors can potentially underlie the diversity in NSPC responses to IR, including age, lineage relationships and regional identity, the latter one due to the fact that the adult VZ/SVZ largely originates from the subpallium, with a more limited contribution from the developing cortex^41^. Surprisingly, the response to irradiation of mouse NSPCs obtained by *in vitro* conversion of pluripotent stem cells appears to be similar to that of adult VZ/SVZ NSPCs, rather than that of foetal cortical NSPCs^20^, but why the irradiation response of *in vitro* generated NSPCs diverges from that of foetus-derived NSPCs remains unclear. Side-by-side comparison of irradiated NSPC cultures of different origins may provide useful insights to unravel these complex questions.

## Supplementary information

Supplementary Information.

## Data Availability

All key data generated or analysed during this study are included in this published article (and its Supplementary Information files). Additional data are available from the corresponding author on reasonable request.
