# Supplementary material for: X-ray irradiated cultures of mouse cortical neural stem/progenitor cells recover cell viability and proliferation with dose-dependent kinetics
Source: Sci Rep. 2020 Apr 16;10:6562. doi: 10.1038/s41598-020-63348-2 (PMC7162981; doi:10.1038/s41598-020-63348-2)
Supplement: Supplementary file 1 — Supplementary Information. [file 41598_2020_63348_MOESM1_ESM.pdf]

X-ray irradiated cultures of mouse cortical neural stem/progenitor cells recover cell viability and proliferation with dose-dependent kinetics

Valerio Licursi[1], Silvia Anzellotti[1], Jessica Favaro[1], Serena Sineri[1], Nicoletta Carucci[1], Enrico Cundari[2], Mario Fiore[2], Giulia Guarguaglini[2], Simone Pippa[1], Paola S. Nisi[1], Fiammetta Vernì[1], Stefano Biagioni[1], Emanuele Cacci[1], Roberto Amendola[3], Giuseppe Lupo[1]*, Rodolfo Negri[1]*

[1]Department of Biology and Biotechnology "C. Darwin", Sapienza University of Rome, Rome, Italy.

[2]Institute of Molecular Biology and Pathology, National Research Council (CNR), Rome, Italy.

[3]Technical Unit for Radiation Biology and Human Health UTBIORAD, ENEA, Rome, Italy.

*corresponding authors: giuseppe.lupo@uniroma1.it, rodolfo.negri@uniroma1.it.

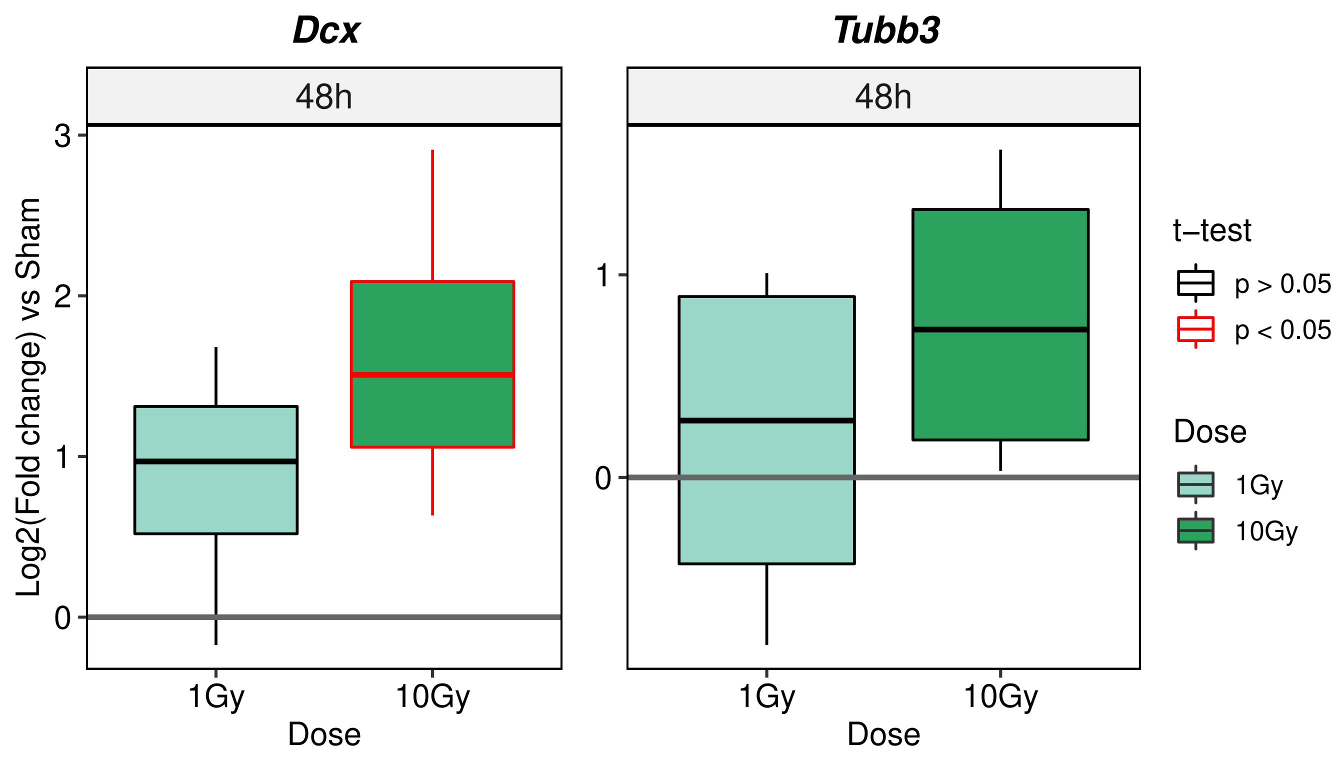

Dcx
Tubb3
48h
48h
Log2(Fold change) vs Sham
3
2
1
0
1
0
1Gy
10Gy
1Gy
10Gy
Dose
Dose
t−test
p > 0.05
p < 0.05
Dose
1Gy
10Gy

Figure S1. X-ray irradiation of mouse cortex NSPC cultures causes a dose-dependent upregulation of neuronal markers

Box-and-whisker plots of the log_2-transformed transcript levels of *Dcx* and *Tubb3* in mouse cortex NSPC cultures at 48h after irradiation with the indicated doses of X-rays, following real-time RT-PCR analysis and normalization to the sham treatment condition. Grey lines represent the relative expression levels in the sham condition. Plots show the results of 4 independent experiments. Plots highlighted in red indicate $p<0.05$ for the comparison between irradiated and sham treated samples according to Student's t-test.

Table S1. Sequences of primers used for real-time RT-PCR assays

Primer	Entrez Gene ID	Sequence
Aldh1l1 Fw	107747	ACCTGCGGATCAAGACTGTG
Aldh1l1 Rv	107747	GGACAGGAGGGTGCTAAGTC
Aqp4 Fw	11829	GAGTCACCACGGTTCATGGA
Aqp4 Rv	11829	CGTTTGGAATCACAGCTGGC
Bax Fw	12028	CACTAAAGTGCCCGAGCTGA
Bax Rv	12028	GAGGCCTTCCCAGCCAC
Cdkn1a (p21) Fw	12575	GATATCCAGACATTCAGAGCCACA
Cdkn1a (p21) Rv	12575	GGGACCGAAGAGACAACGG
Dcx Fw	13193	CCAGCAGTCAGCTCTCAACA
Dcx Rv	13193	ATGGAATCGCCAAGTGAATC
Gadd45a Fw	13197	TGGTGACGAACCCACATTCA
Gadd45a Rv	13197	ACCCACTGATCCATGTAGCG
Gapdh Fw	14433	CTGCCCAGAACATCATCCCT
Gapdh Rv	14433	ACTTGGCAGGTTTCTCCAGG
Gfap Fw	14580	CGAAGAAAACCGCATCACCAT
Gfap Rv	14580	GGCCTTCTGACACGGATTTG
linc-p21 Fw	100504267	TGGAAAACTGGGCCAACAGT
linc-p21 Rv	100504267	CTCTGCCAGTGTCCAGGAAG
Nes Fw	18008	CAACTGGCACACCTCAAGAT
Nes Rv	18008	AGGTGTCTGCAAGCGAGAGT
Rpl19 Fw	19921	AGACCAAGGAAGCACGAAAG
Rpl19 Rv	19921	GCCGCTATGTACAGACACGA
Rpl29 Fw	19944	GCCTAAGGTCCAAACCAAGG
Rpl29 Rv	19944	TGTCTTCACACTGGCAGGAG
Tubb3 Fw	22152	CAATGAGGCCTCCTCTCACAA
Tubb3 Rv	22152	TCCATGGTTCCAGGTTCCAA

Table S2. Statistical Tables

Two-way ANOVA with Tukey's HSD test Figure 1

Only comparisons between samples at the same point with p < 0.05 are shown

Comparison	Mean Difference	C.I. low	C.I. high	Adjusted p value
10 Gy 24h-1 Gy 24h	0.16	0.02	0.31	$p < 0.05$
Sham 24h-10 Gy 24h	-0.22	-0.37	-0.08	$p < 0.001$
Sham 48h-10 Gy 48h	-0.22	-0.43	-0.02	$p < 0.05$

Two-way ANOVA with Tukey's HSD test Figure 2

Only comparisons between irradiated and sham treated samples at the same point with p < 0.05 are shown

G1 phase analysis

Comparison	Mean Difference	C.I. low	C.I. high	Adjusted p value
Sham 24h-1 Gy 24h	-11.00	-19.45	-2.55	$p < 0.01$
Sham 24h-10 Gy 24h	-13.75	-22.86	-4.64	$p < 0.0001$
Sham 48h-10 Gy 48h	-12.67	-25.04	-0.29	$p < 0.05$
Sham 8h-10 Gy 8h	17.25	7.97	26.53	$p < 0.0001$

S phase analysis

Comparison	Mean Difference	C.I. low	C.I. high	Adjusted p value
Sham 8h-0.2 Gy 8h	9.38	2.68	16.07	$p < 0.001$
Sham 24h-1 Gy 24h	7.24	1.14	13.35	$p < 0.01$
Sham 8h-1 Gy 8h	12.63	5.93	19.32	$p < 0.0001$
Sham 24h-10 Gy 24h	15.69	9.12	22.27	$p < 0.0001$
Sham 48h-10 Gy 48h	14.00	5.07	22.93	$p < 0.0001$
Sham 8h-10 Gy 8h	16.63	9.93	23.32	$p < 0.0001$

G2/M phase analysis

Comparison	Mean Difference	C.I. low	C.I. high	Adjusted p value
Sham 8h-1 Gy 8h	-6.38	-12.13	-0.62	$p < 0.05$
Sham 8h-10 Gy 8h	-33.88	-39.63	-28.12	$p < 0.0001$

Two-way ANOVA with Tukey's HSD test Figure 3

Only comparisons with p < 0.05 are shown

Comparison	Mean Difference	C.I. low	C.I. high	Adjusted *p* value
10 Gy 2h-10 Gy 24h	681.50	250.16	1112.83	$p < 0.01$
10 Gy 5h-10 Gy 24h	509.48	78.15	940.82	$p < 0.05$
Sham 24h-10 Gy 2h	-809.74	-1241.07	-378.40	$p < 0.001$
Sham 2h-10 Gy 2h	-781.15	-1212.48	-349.81	$p < 0.001$
Sham 5h-10 Gy 2h	-813.85	-1245.19	-382.51	$p < 0.001$
Sham 24h-10 Gy 5h	-637.72	-1069.06	-206.39	$p < 0.01$
Sham 2h-10 Gy 5h	-609.13	-1040.47	-177.80	$p < 0.01$
Sham 5h-10 Gy 5h	-641.84	-1073.17	-210.50	$p < 0.01$

One-sample Student's t-test Figure 4

Data used for statistical analysis were obtained as the ratio between irradiated and sham treated samples for each time point - only p-values < 0.05 are shown

Gene	Sample	Mean	C.I. low	C.I. high	Alt. Hypothesis	*p* value
linc-p21	0.2Gy 4h	0.51	0.03	0.98	two sided	P < 0.05
linc-p21	1Gy 4h	0.53	0.31	0.75	two sided	P < 0.001
linc-p21	10Gy 4h	1.17	0.59	1.76	two sided	P < 0.01
linc-p21	1Gy 8h	0.66	0.22	1.10	two sided	P < 0.05
linc-p21	10Gy 8h	0.78	0.32	1.24	two sided	P < 0.05
linc-p21	1Gy 24h	0.96	0.57	1.34	two sided	P < 0.001
linc-p21	10Gy 24h	2.11	1.17	3.05	two sided	P < 0.01
linc-p21	10Gy 48h	1.64	0.21	3.06	two sided	P < 0.05
Cdkn1a	0.2Gy 4h	0.88	0.47	1.28	two sided	P < 0.01
Cdkn1a	1Gy 4h	1.01	0.72	1.30	two sided	P < 0.0001
Cdkn1a	10Gy 4h	1.69	0.38	3.00	two sided	P < 0.05
Cdkn1a	1Gy 8h	1.59	0.21	2.97	two sided	P < 0.05
Cdkn1a	10Gy 8h	1.77	0.53	3.01	two sided	P < 0.05
Cdkn1a	1Gy 24h	1.10	0.73	1.47	two sided	P < 0.0001
Cdkn1a	10Gy 24h	2.24	0.74	3.74	two sided	P < 0.05
Cdkn1a	10Gy 48h	2.21	1.26	3.16	two sided	P < 0.01
Bax	0.2Gy 4h	0.16	0.01	0.31	two sided	P < 0.05
Bax	1Gy 4h	0.48	0.30	0.67	two sided	P < 0.001
Bax	10Gy 4h	0.75	0.49	1.00	two sided	P < 0.01
Bax	1Gy 8h	1.12	0.08	2.15	two sided	P < 0.05
Bax	10Gy 8h	1.03	0.50	1.56	two sided	P < 0.05

Bax	0.2Gy 24h	0.09	0.02	0.17	two sided	P < 0.05
Bax	1Gy 24h	0.48	0.19	0.78	two sided	P < 0.01
Bax	10Gy 24h	1.03	0.38	1.68	two sided	P < 0.05
Bax	10Gy 48h	1.30	0.42	2.18	two sided	P < 0.05
Gadd45a	1Gy 4h	0.43	0.15	0.71	two sided	P < 0.01
Gadd45a	10Gy 8h	0.67	0.26	1.09	two sided	P < 0.05
Gadd45a	10Gy 24h	1.34	0.27	2.42	two sided	P < 0.05
Gadd45a	10Gy 48h	2.24	1.61	2.86	two sided	P < 0.01

One-sample Student's t-test Figure 5

Data used for statistical analysis were obtained as the ratio between irradiated and sham treated samples for each time point - only p-values < 0.05 are shown

Gene	Sample	Mean	C.I. low	C.I. high	Alt. Hypothesis	*p* value
Nes	10Gy 48h	0.44	0.14	0.75	two sided	P < 0.05
Gfap	1Gy 24h	1.19	0.02	2.36	two sided	P < 0.05
Gfap	10Gy 24h	2.00	0.31	3.68	two sided	P < 0.05
Gfap	1Gy 48h	1.11	0.18	2.04	two sided	P < 0.05
Gfap	10Gy 48h	3.67	2.53	4.82	two sided	P < 0.01
Gfap	10Gy 8d	1.17	0.43	1.91	two sided	P < 0.01
Aldh1l1	10Gy 48h	1.71	0.89	2.52	two sided	P < 0.01

One-sample Student's t-test Figure S1

Data used for statistical analysis were obtained as the ratio between irradiated and sham treated samples for each time point - only p-values < 0.05 are shown

Gene	Sample	Mean	C.I. low	C.I. high	Alt. Hypothesis	*p* value
Dcx	10Gy 48h	1.64	0.09	3.19	two sided	P < 0.05
